# Evaluation of methods for the reduction of contaminating host reads when performing shotgun metagenomic sequencing of the milk microbiome

**DOI:** 10.1038/s41598-020-78773-6

**Published:** 2020-12-10

**Authors:** Min Yap, Conor Feehily, Calum J. Walsh, Mark Fenelon, Eileen F. Murphy, Fionnuala M. McAuliffe, Douwe van Sinderen, Paul W. O’Toole, Orla O’Sullivan, Paul D. Cotter

**Affiliations:** 1 Teagasc Food Research Centre, Moorepark, Fermoy, Co. Cork Ireland; 2 School of Microbiology, University College Cork, Cork, Ireland; 3 APC Microbiome Ireland, Cork, Ireland; 4 Precision Biotics, Cork, Ireland; 5 UCD Perinatal Research Centre, School of Medicine, University College Dublin, National Maternity Hospital, Dublin, Ireland

**Keywords:** Next-generation sequencing, Metagenomics, Metagenomics

## Abstract

Shotgun metagenomic sequencing is a valuable tool for the taxonomic and functional profiling of microbial communities. However, this approach is challenging in samples, such as milk, where a low microbial abundance, combined with high levels of host DNA, result in inefficient and uneconomical sequencing. Here we evaluate approaches to deplete host DNA or enrich microbial DNA prior to sequencing using three commercially available kits. We compared the percentage of microbial reads obtained from each kit after shotgun metagenomic sequencing. Using bovine and human milk samples, we determined that host depletion with the MolYsis complete5 kit significantly improved microbial sequencing depth compared to other approaches tested. Importantly, no biases were introduced. Additionally, the increased microbial sequencing depth allowed for further characterization of the microbiome through the generation of metagenome-assembled genomes (MAGs). Furthermore, with the use of a mock community, we compared three common classifiers and determined that Kraken2 was the optimal classifier for these samples. This evaluation shows that microbiome analysis can be performed on both bovine and human milk samples at a much greater resolution without the need for more expensive deep-sequencing approaches.

## Introduction

Milk is an important source of nutrition for both humans and animals. Human breast milk contains bioactive compounds that contribute to a child’s development and growth^1^. With the development of culture-independent methods for studying the microbiome, increasing interest has been devoted to understanding the interplay between breast milk and the infant gut microbiome^2^. Indeed infants primarily fed human breast milk within the first few months of life have been shown to harbour a distinct microbiome from that of non-breast-fed infants^3^. 16S rRNA gene amplicon-based analysis of the human milk microbiome has also improved the understanding of the bacterial community related to mastitis. Despite these advances, the application of shotgun metagenomic approaches has the potential to provide even greater insights into the milk microbiome to help with prevention or treatment of lactational mastitis and further promote infant and maternal health^4^.

Besides human milk, animal milk from cows, goats, sheep, and buffalo and the resultant dairy products, have proven to be versatile and valuable foods commonly consumed by humans^5–7^. The bovine milk microbiome has been intensely studied, with a view to assessing and improving animal health and ensuring product quality and safety for human consumption^8–10^. As with human mastitis, culture-based microbiology methods have had variable success in the diagnosis of bovine mastitis due to their inability to detect low abundance pathogens and because of challenges associated with subclinical infection of unknown aetiology^11^. Sequencing-based approaches have also addressed this issue to some degree^12^. In addition to animal health, research on the bovine milk microbiome has been performed motivated by the near-universal use of milk as an ingredient in many dairy products worldwide. Although certain bacteria, when present in milk products, can improve the resulting sensory and flavour properties^13^, other microorganisms can negatively influence milk by deteriorating its quality, causing spoilage, or making it unsafe for consumption^14^.

High-throughput sequencing technologies, both targeted amplicon sequencing, e.g. 16S rRNA gene, and shotgun metagenomics, have been widely adopted for the study of microbiomes, including relatively highly diverse and species-rich microbiomes such as the human gut^15–17^, human oral cavity^18^, and soil^19,20^. For environments with a low microbial load and diversity such as milk, amplicon sequencing has been the primary tool used. For bovine milk analysis, this has provided insights into the compositional changes in raw milk due to environmental factors, processing or the production of cheese^1,10,21–23^. Notwithstanding the value of data already generated, extraction methodologies have been identified as having an impact on downstream sequencing data from 16S rRNA sequencing studies^24,25^. Furthermore, although these amplicon sequencing-based approaches have provided valuable insights, shotgun metagenomic sequencing can provide greater taxonomic insights, particularly with regard to elucidating the functional potential of the microbes present and the ability to generate metagenome-assembled genomes (MAGs)^26^. These MAGs provide essentially-complete genome information for key taxa present in the sample, revealing their functional and safety related properties^27^. However, shotgun sequencing, as an untargeted approach, sequences all the DNA in the sample, including that from the human or animal host, which in the case of milk represents a considerable majority (up to 95%) of the DNA present^28^. To address this challenge, previous shotgun metagenomic-based studies of the human milk microbiome^1,29^ carried out sequencing at great depth, followed by post-processing of data to remove non-microbial reads using bioinformatic approaches. In addition to generating large numbers of reads that need to be discarded, the limited number of microbial reads generated prevents the detection of low abundance species^30^.

Many recent studies involving clinical samples with high levels of host DNA, such as saliva, sputum and joint fluid, have investigated depleting host DNA through commercially available kits or other chemical methods^31–34^. However, such approaches have not been applied to milk. Often due to logistics or sampling circumstances, samples are frozen and stored before DNA is extracted. To address this technological caveat, the aim of this study was to evaluate methods of host DNA depletion/microbial DNA enrichment to optimise shotgun sequencing-based analysis of the milk microbiome.

## Results

### Efficiency of methods for improving the proportion of microbial reads

**Figure 1:**
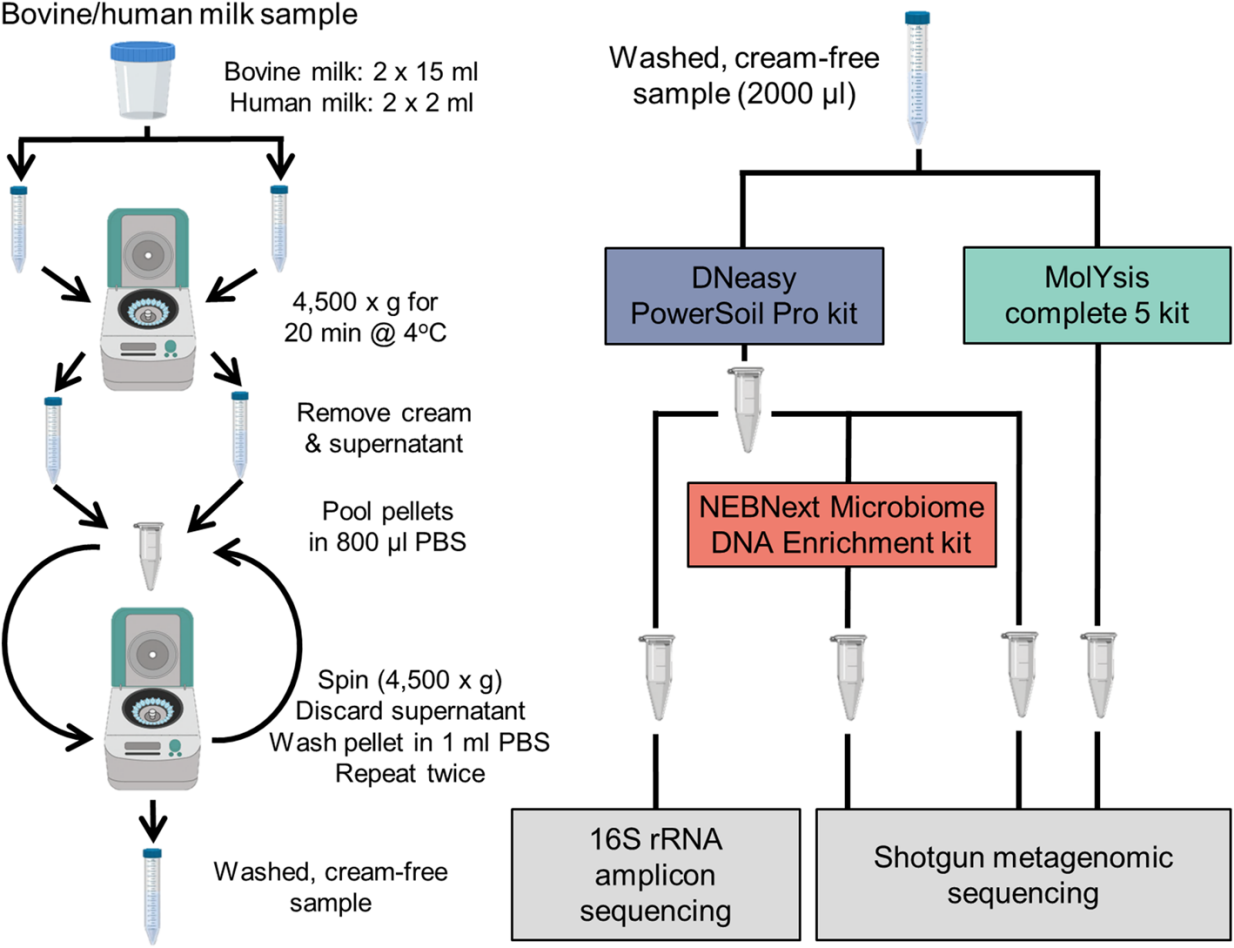
Experimental procedure used for the study. Process for skimming both bovine and human milk samples is indicated on the left. Direct extraction, host depletion, or microbial enrichment approaches are indicated on the right. This figure was created in part with BioRender.com.

**Figure 2:**
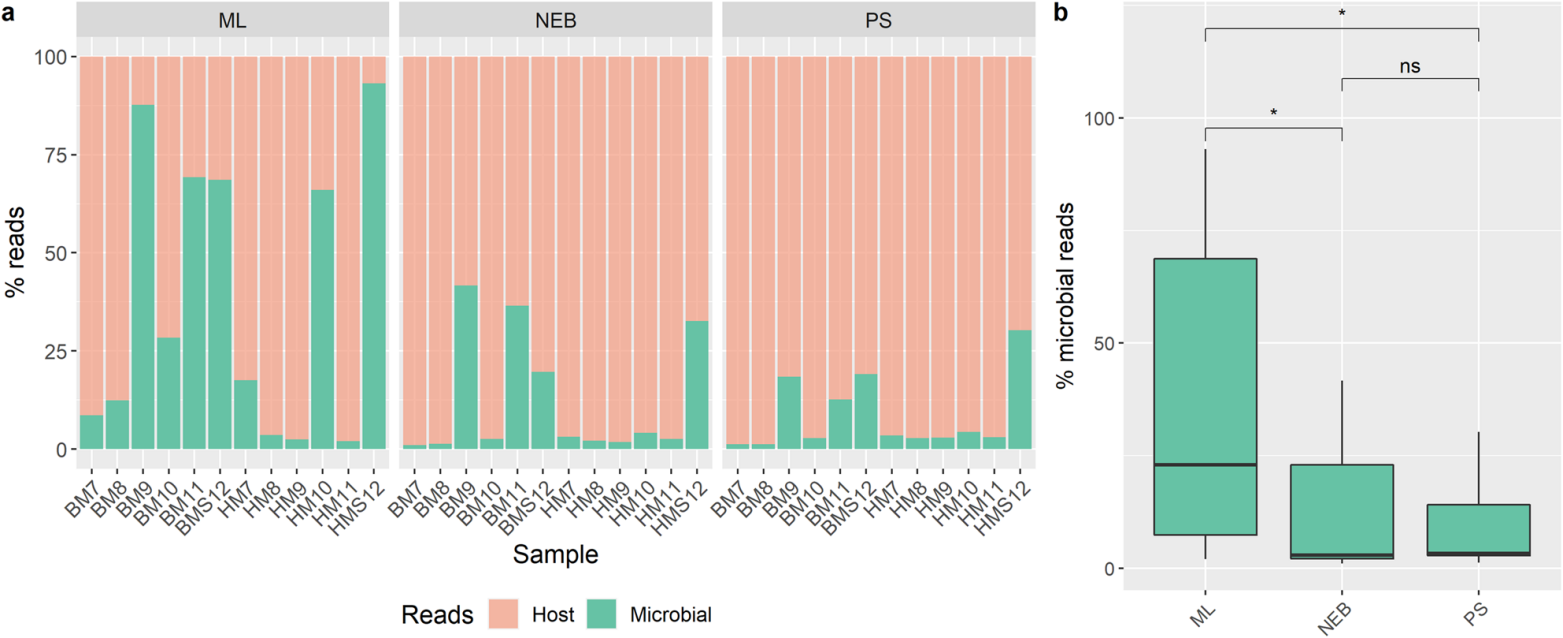
ML kit produced significantly higher proportions of microbial reads. (**a**) Host and microbial reads of bovine (BM) and human (HM) milk samples processed using three different methods; MolYsis complete5 kit (ML), NEBNext Microbiome Enrichment kit (NEB) and QIAGEN DNeasy PowerSoil Pro kit (PS). (**b**) Comparison of the mean microbial reads of all samples within one extraction method to another. Asterisk denotes significant differences of *p* value < 0.05 as determined using Student’s *t *test. Figures were produced using R^48^.

Microbial DNA was extracted from each of six bovine and six human milk samples using the three different methods (Fig. 1). Seven approaches were provisionally tested to assess their suitability for extracting DNA from low volumes of material (Supplementary Figure S1). From these, only the DNeasy PowerSoil Pro (PS) and MolYsis complete5 (ML) kits yielded sufficient DNA concentrations. Therefore, the three methods evaluated were (1) the PS kit, which is commonly used in microbiome studies and is not specifically designed to enrich microbial DNA or deplete host DNA, (2) the PS approach but used in combination with the NEBNext Microbiome Enrichment kit (NEB) and (3) ML kit, designed to decrease the amount of host DNA present. The extracted DNA was subjected to shotgun metagenomic sequencing, generating an average of 3,972,773 (± 383,371) high quality paired end reads per sample (Supplementary Table S1), Although the DNA extraction approach taken did not significantly impact on the total number of reads per sample, the proportions of host to microbial reads differed considerably (Fig. 2a). More specifically, the ML kit yielded a significantly higher (P < 0.05) % of microbial reads (Fig. 2b), i.e., 38.31% average (range 2.01–93.12%), relative to the other two methods, i.e., NEB kit 12.45% average, (range 1.03–41.63%) and PS kit 8.54% average (range 1.22–30.28%).

### Differential performance of taxonomic classification algorithms on spiked samples

Bovine and human milk samples (BMS12 and HMS12) were spiked with a mock community of ten strains, present at equal proportions, to investigate the potential for the introduction of bias due to differences in extraction efficiencies from different bacteria and to inform the choice of bioinformatic classification tools. Negative extraction controls were also included to investigate the potential for kit contamination.

**Figure 3:**
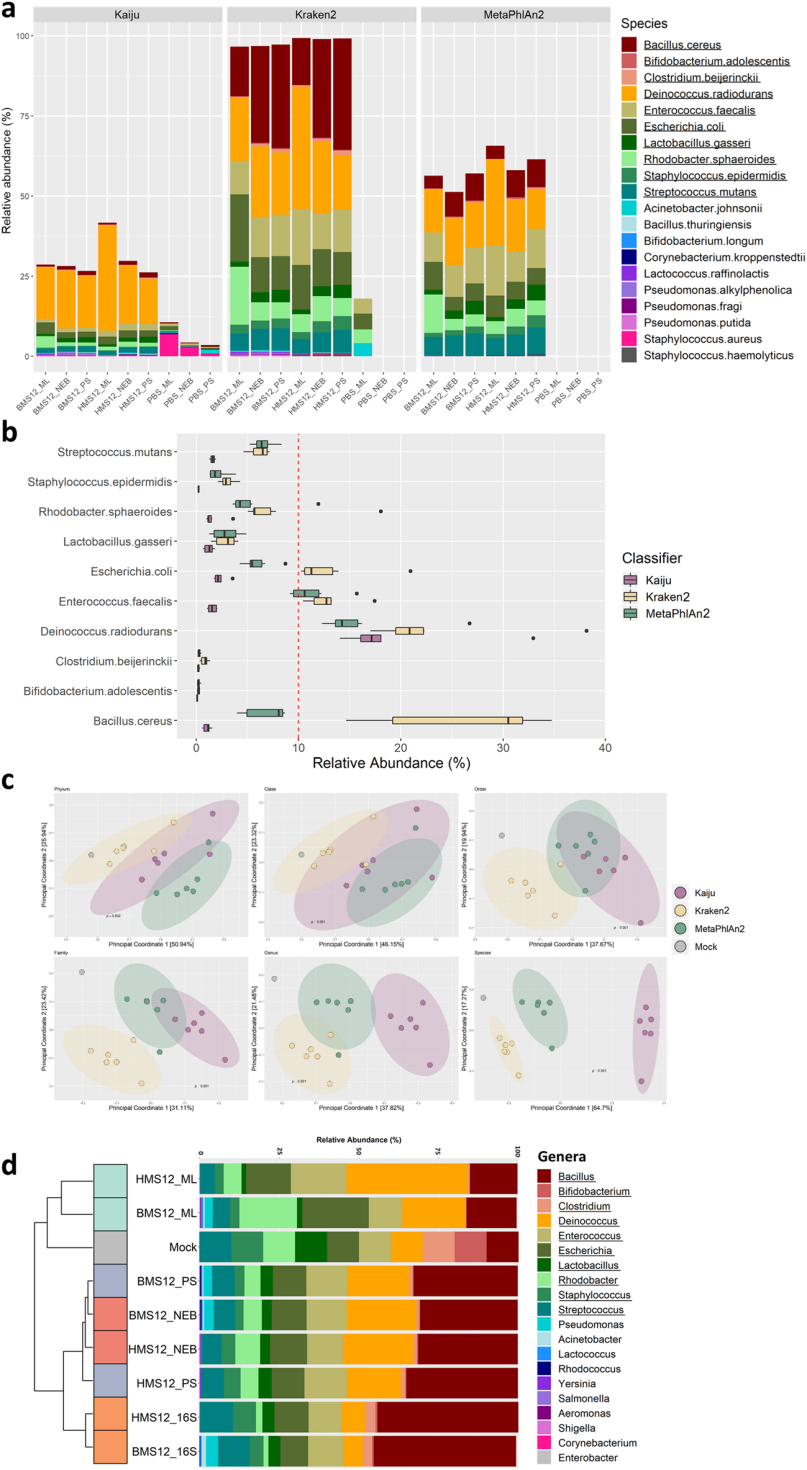
Mock community samples impacted by choice of bioinformatics classifier rather than method. (**a**) Top 20 species by relative abundance of non-host reads are displayed as determined by three different taxonomic classifiers. Unclassified and low abundance species account for the non-displayed reads. The species underlined are those present in the spiked mock community. (**b**) Comparison of the relative abundances of the 10 species in the mock community in the spiked milk samples according to each classification tool. Red dotted line indicates the expected 10% mark. (**c**) Bray–Curtis dissimilarity plots of all 6 spiked mock community samples, coloured by classifier, for each taxonomic rank. The expected mock community composition is in grey. Significant differences are noted with their *p* value as calculated using ADONIS. (**d**) Top 20 genus level microbial composition of each spiked milk community as determined by both shotgun metagenomics as classified using Kraken2 and 16S rRNA amplicon sequencing. Genera underlined are those present in the spiked mock community. Samples are clustered based on Bray–Curtis dissimilarity with expected mock community included. Figures were produced using R^48^.

After sequencing and quality control of the sequences, taxonomic classification was performed using three different tools, Kaiju, Kraken2, and MetaPhlAn2. Overall, the top 20 species detected accounted for 48.7% of the species identified by Kaiju, 98.3% of the species identified by Kraken2, and 61.3% of the species identified by MetaPhlAn2 (Fig. 3a). The mock community accounted for an average relative abundance of 29.2% of the species identified by Kaiju, 96.6% of the species classified by Kraken2 and 58.1% of the species identified by MetaPhlAn2. All classifiers detected the 10 mock community species, although at varying relative abundances. In general, all three tools showed an under-representation of *Bifidobacterium adolescentis* (0.03–0.49%) and *Clostridium beijerinckii* (0.08–1.04%) in samples and an over-representation of *Deinococcus radiodurans* (12.29–38.16%) (Fig. 3a,b). Both Kraken2 and MetaPhlAn2 produced similar mock community composition profiles, albeit with Kraken2 assigning a much higher % of reads. Both Kaiju and Kraken2 assigned reads within the negative control samples, while MetaPhlAn2 only assigned reads from one of the three negative control samples. The taxa identified were distinct from those detected in milk samples (without spiked controls) (Supplementary Table S2).

When the abundances of the 10 mock community strains were investigated in greater detail, Kraken2 and MetaPhlAn2 were found to assign the strains at relative abundances closer to that expected for the mock community than Kaiju. Kraken2 identified *Escherichia coli*, *Enterococcus faecalis*, *D. radiodurans,* and *Bacillus cereus* at a higher abundance than expected, while MetaPhlAn2 appeared to over-represent both *E. faecalis* and *D. radiodurans* (Fig. 3b). When the Bray–Curtis dissimilarity of all mock community samples were plotted in individual PCoA plots by taxonomic rank, the overall dissimilarity increased further down the taxonomic ranks, but communities classified with Kraken2 consistently clustered closer to the mock community from phylum to species (Fig. 3c). At the highest rank (phylum), Kraken2 clustered closest to the mock community and significant differences were observed between the Kraken2 and MetaPhlAn2 communities (*p* = 0.012). Ultimately, Kraken2 was selected for further downstream analysis as it performed best in terms of overall correct assignment of mock community DNA.

The spiked mock community samples were additionally compared using Kraken2-classified metagenomic reads with 16S rRNA amplicon reads. The determined community composition, using these two different sequencing approaches, was similar, with small differences in relative abundances (Fig. 3d). Additional genera, including *Acinetobacter* and *Lactococcus* were consistently detected in bovine milk samples regardless of sequencing method.

### Analysis of bovine and human milk samples—community structure and taxonomic and functional profiles

**Figure 4:**
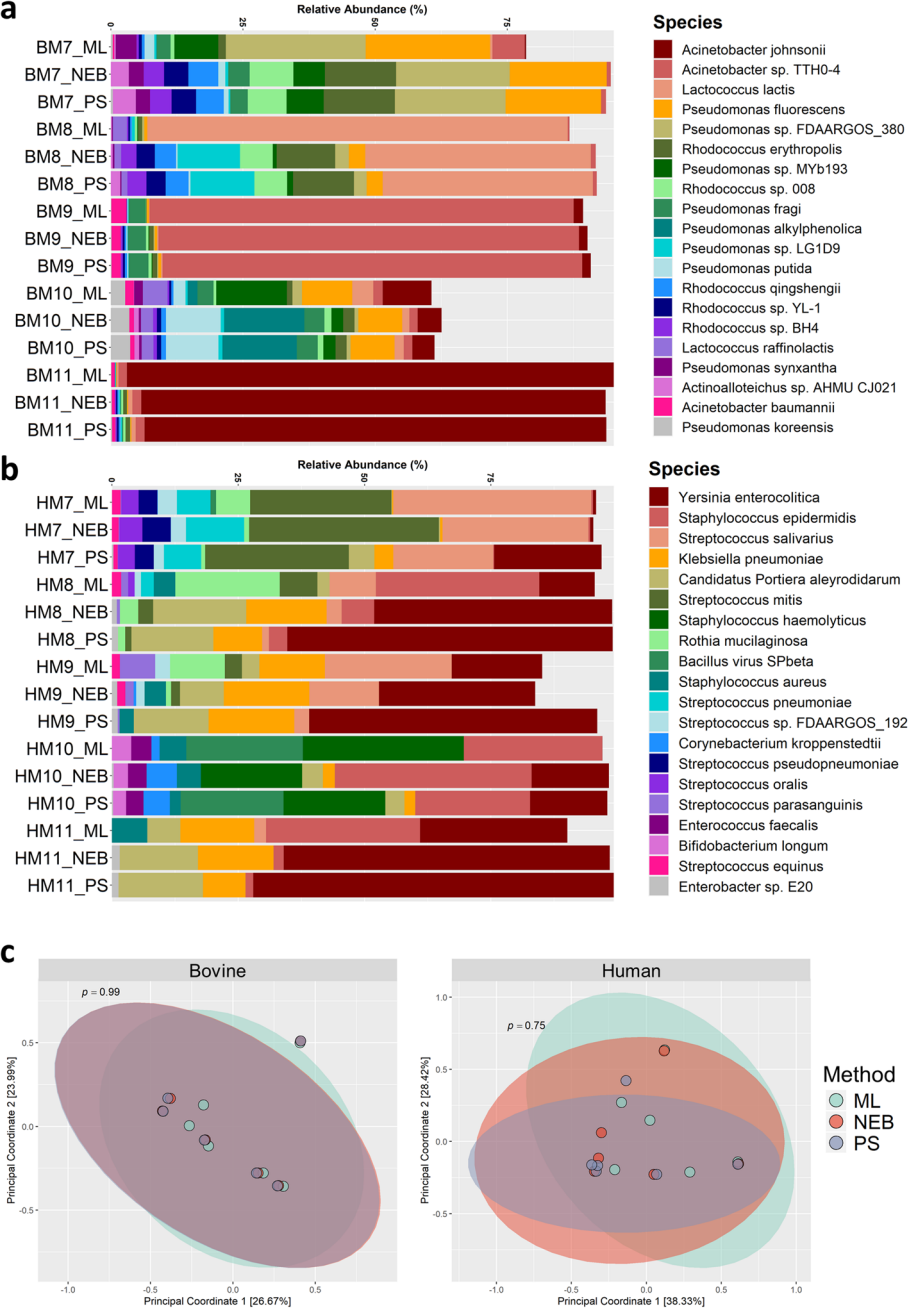
Both the bovine and human milk samples each had similar species level composition and community diversity. Top 20 species composition of all test bovine (**a**) and human (**b**) milk samples using all three extraction methods and clustered based on Bray–Curtis dissimilarity. (**c**) Bray–Curtis dissimilarity plots for both bovine (left) and human (right) milk samples for microbial communities determined using all three methods. Statistical community dissimilarities were calculated using ADONIS. Figures were produced using R^48^.

The species-level composition of bovine and human milk microbiome samples as assigned by Kraken2 was compared across the different extraction methods used (Fig. 4a). In bovine milk, aliquots of the same samples that were extracted using different methods in general had generated similar profiles, albeit with slightly varying relative abundances. However, ML extraction from BM7 led to the additional assignment of reads to *Lactococcus lactis*, *Pseudomonas alkylphenolica* and *Acinetobacter baumannii* at low abundances (< 0.1%). For sample BM8, *L. lactis* was detected at higher relative abundances in the sample extracted with the ML kit (compared to the PS and NEB kit).

With regard to the human milk samples (Fig. 4b), apart from HM7, the relative taxonomy of each sample varied between by extraction methods. HM8_ML was found to be more similar in species composition to HM10_NEB and HM11_ML, reflecting the detection of *Staphylococcus epidermidis* in these samples. Similarly, a high relative abundance of *Yersinia enterocolitica* was detected in the HM9_PS, HM8_NEB, HM8_PS, HM11_NEB and HM11_PS samples.

Despite the taxonomic compositional differences, no significant dissimilarity was observed between the methods for either bovine (ADONIS: *p* = 0.997, R^2^ = 0.02362) or human (ADONIS: *p* = 0.749, R^2^ = 0.08218) samples (Fig. 4c). The overall community diversity was not impacted by any method used, implying that there was no community-level bias introduced by any of the methods.

In terms of functional profiling, the predicted microbiome function in bovine or human milk samples using the three sub-domains of gene ontology (cellular components, biological processes and molecular functions) was not significantly impacted by extraction method used (Supplementary Figure S2).

### Characterization of the milk microbiome

**Figure 5:**
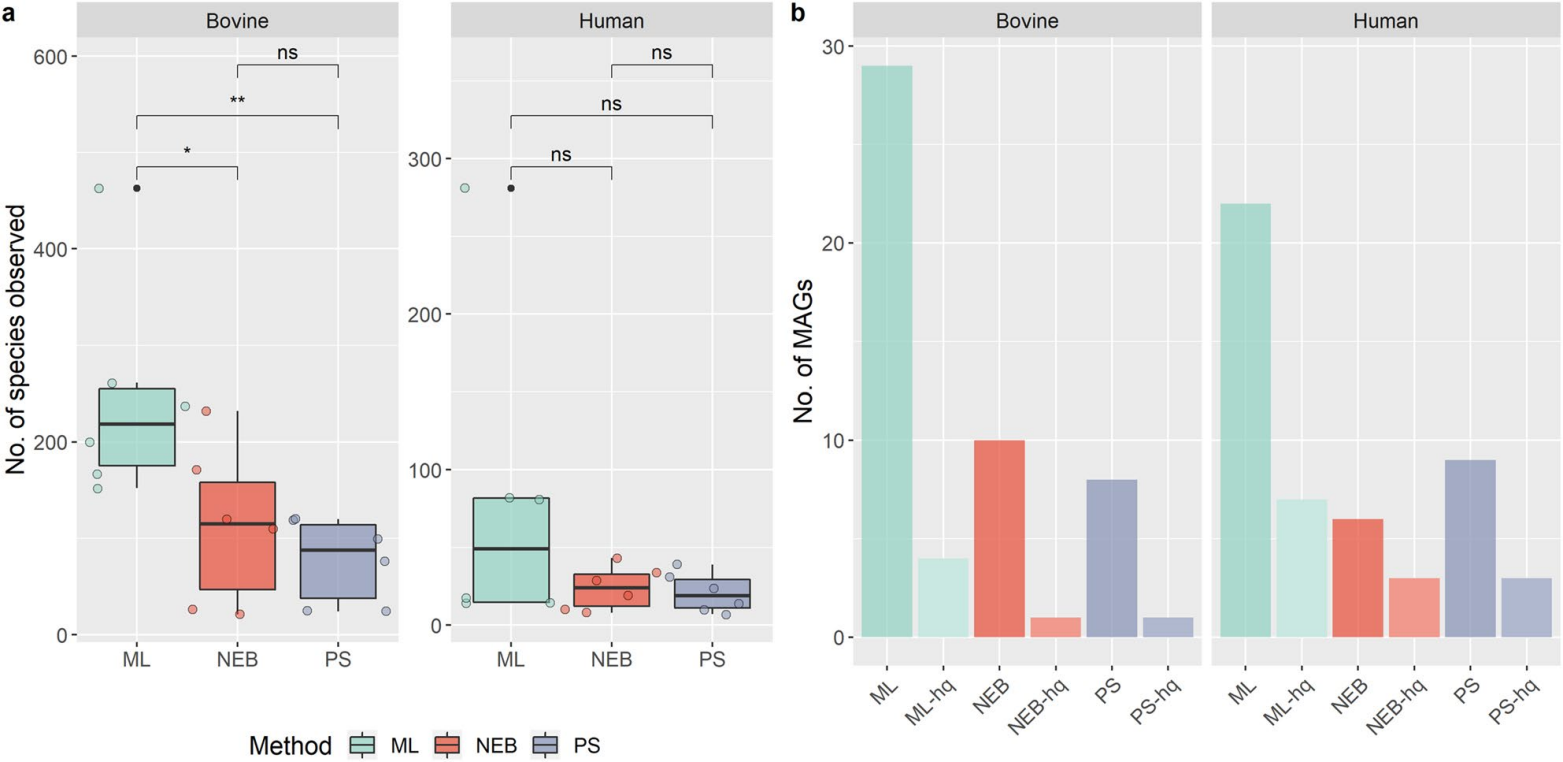
ML kit generated higher numbers of observable species and metagenome assembled genomes in milk samples. (**a**) Total number of species detected by each method in either bovine (left) or human (right) milk samples. A single or double asterisk indicates a significant (P < 0.05) or highly significant (P < 0.01) difference between species number as determined by ANOVA. (**b**) The number of total and high-quality MAGs (hq) recovered from sequenced samples using either method. High-quality MAGs were greater than 90% completeness and less than 5% contamination. Figures were produced using R^48^.

Extraction of DNA from milk samples using the ML kit resulted in greater apparent species richness from an overall higher detection of unique observed species. This increase was significant (p < 0.05) among bovine milk samples (Fig. 5a). A total of 84 metagenome-assembled genomes (MAGs) were recovered across all samples, of which 19 were considered high quality, using the cut-off of > 90% completeness and < 5% contamination (Fig. 5b, Table 1). From among these 19, 11 were assembled with sequence data derived from DNA extracted using the ML kit, while only 4 high-quality MAGs were recovered from DNA extracted from each of the other two methods. The majority of the high-quality MAGs recovered were from milk samples with the spiked mock community but *Corynebacterium pyruviciproducens* and *E. faecalis* MAGs were also recovered from the HM10 sample extracted using the ML kit. To evaluate if the differences between the direct sequencing (PS) and the depletion (ML) approach were due to the increased read depth or kit-introduced bias, we subsampled 50,000 non-host reads from each sample five times. Bray–Curtis analysis revealed that there was no significant difference in the community structure between both bovine (ADONIS: *p* = 0.15, R^2^ = 0.0260) and human (ADONIS: *p* = 0.07, R^2^ = 0.0439) samples sequenced using either approach (Supplementary Figure S3a,b). When comparing the dispersion within samples all related bovine samples were similar between kits, one related human sample was significantly different, and one sample could not be compared after subsampling (Supplementary Figure S3c,d).

**Table 1:** High quality metagenome-assembled genomes from shotgun sequencing analysis.

Sample	Kit	Taxonomy	Completeness (%)^a^	Contamination (%)^b^
HM10_ML	ML	*Corynebacterium pyruviciproducens*	99.76	0.1
BMS12_ML	ML	*Streptococcus mutans*	99.63	0
HMS12_ML	ML	*Enterococcus faecalis*	99.63	0
HMS12_ML	ML	*Deinococcus radiodurans*	99.15	0.21
HMS12_ML	ML	*Streptococcus mutans*	98.88	0
BMS12_ML	ML	*Deinococcus radiodurans*	98.72	0.21
HM10_ML	ML	*Enterococcus faecalis*	98.5	0
BMS12_ML	ML	*Bacillus cereus*	98.3	0.33
BMS12_ML	ML	*Rhodobacter sphaeroides*	97.58	0.15
HMS12_ML	ML	*Bacillus cereus*	96.18	0.42
HMS12_ML	ML	*Rhodobacter sphaeroides*	92.05	0.25
HMS12_NEB	NEB	*Deinococcus radiodurans*	96.17	0.21
HMS12_NEB	NEB	*Enterococcus faecalis*	94.88	0.94
HMS12_NEB	NEB	*Bacillus cereus*	94.32	0.33
BMS12_NEB	NEB	*Deinococcus radiodurans*	90.92	0.21
HMS12_PS	PS	*Bacillus cereus*	97.99	0.33
HMS12_PS	PS	*Streptococcus mutans*	96.38	1.06
HMS12_PS	PS	*Deinococcus radiodurans*	95.96	0
BMS12_PS	PS	*Deinococcus radiodurans*	91.9	0.64

^a^Completeness: ratio of observed single-copy marker genes to total single-copy marker genes in the chosen marker gene set^46^.

^b^Contamination: ratio of observed single-copy marker genes in ≥ 2 copies to total single-copy marker genes in the chosen marker gene set^46^.

## Discussion

Shotgun metagenomic sequencing is a powerful method for the study of many different microbiome types. In order to maximise the potential of this method for characterizing specific microbial populations, the removal of host DNA is necessary to allow for a greater sequencing depth of the microbial populations of interest. The present study evaluated three different approaches for their efficiency in host DNA depletion and, ultimately, their effectiveness with respect to studying the milk microbiome. Results showed that the ML kit gave significantly higher percentages of microbial reads when compared with the NEB kit and PS kit. The ML kit uses a chaotropic buffer that selectively lyses host cells, followed by the degradation and removal of the released host DNA using DNAse. The microbial DNA in the samples remains intact before being subjected to DNA extraction. This contrasts with the NEBNext Microbiome DNA Enrichment kit, which exploits the methylation of DNA at CpG sites in eukaryotic cells, using magnetic beads to selectively bind and remove CpG-methylated host DNA. Studies using the NEB kit for analysis of other microbiomes have shown varying results^31,33,34^. The protocol recommends an input of high molecular weight gDNA (≥ 15 kb for optimal performance) and this was not possible to achieve in the milk samples due to the limited sample volume, and their lower relative microbial abundance when compared to samples such as human stool. Thus, the findings provided here relate to this method’s performance in relation to milk samples only and should not be extrapolated to other sample types. The PS kit and similar kits have been widely used in various microbiome studies^10,22,35,36^. The PS kit uses chemical and mechanical lysis before elution using a silica membrane spin column. Although it has no specific step for the depletion of host DNA, it has been found to be effective in terms of total DNA yield, which was evident in this study (Supplementary Table S1)^35^.

In this study, we found that the choice of bioinformatics tool for classification had a greater influence on community composition than the method of DNA extraction, as determined with the mock community analysis (Figs. 3, 4). Kraken2 and Kaiju are taxonomic binning tools, based on alignments with reference genomes. Kraken2, which was the tool that was ultimately chosen for downstream analysis, is a nucleotide sequence classifier that assigns taxonomic labels to DNA sequences. It uses an algorithm that relies on exact *k*-mer matches that records the species identifier for every *k*-mer in every genome. To classify a sequence, each *k*-mer in the sequence is mapped to the lowest-common ancestor (LCA) of the genomes that contain that *k*-mer in a database^37^. Kaiju works similar to Kraken2 except by classifying reads using a reference database comprising the annotated protein-coding genes of a set of microbial genomes. However, unlike Kraken2, Kaiju performed poorly in classifying the mock community samples in this study. MetaPhlAn2, unlike Kraken2 and Kaiju, is based on the alignments with species-specific marker gene sequences. Quality-controlled reads are mapped to a database of unique clade-specific marker genes with high discriminatory power, which are more optimized for human-associated gut microbial communities^38^. The relative abundances of each clade in the samples are then estimated with species-level resolution^39^. As MetaPhlAn2 is based on marker genes, considerable microbial sequencing depth is needed to be able to detect marker genes from low abundance organisms^40^. Although MetaPhlAn2 has been found to be sensitive for low complexity food microbiomes^41^, its performance in this study to characterize the milk microbiome was not as efficient as Kraken2, particularly in terms of total assignment, which was lower for samples classified using MetaPhlAn2 compared to Kraken2. Taken together, these results confirm that the choice of bioinformatic tool has an important influence on taxonomic composition, particularly in low abundance environments. The impact of choice of classification tools have already been reported in other studies^41^.

The comparison of the methods used in this study showed that there were no biases in taxonomic composition and community structure when any method was used. Although the increased microbial sequencing depth of the ML kit allowed for the identification of more unique species from the milk samples compared to the other two methods, there were no significant differences in beta diversity (Fig. 4c). Similar results were found in the analysis of functional data of the samples, where no particular method generated functional profiles that were distinct or significantly different (Supplementary Figure S2). In addition, we showed that metagenomic sequencing of milk samples is not limited in identification of taxa compared to the standard method of amplicon sequencing (Fig. 3d).

The ability to track microbes at strain level is of great importance and interest both in the food industry and in uncovering mother-to-infant microbial transfer^22,29^. The ability to generate high-quality MAGs from the ML processed samples is promising when considering this challenge. Apart from recovery of mock community strains, the increased microbial sequencing depth allowed for the assembly of high-quality *Corynebacterium pyruviciproducens* and *Enterococcus faecalis* genomes from the metagenome of a human milk sample. This suggests that with greater microbial sequencing depth, the recovery of high-quality MAGs can uncover species not previously identified in the milk microbiome, particularly for species that are difficult to culture.

This study aimed to evaluate three different methods for improved microbial read sequencing depth, however certain limitations remain. The study was performed using a small number of samples, yet the current sample size was sufficient to allow for the identification of significant differences between methods. Additionally, the microbial or host DNA concentration was not absolutely quantified, which could provide a greater insight into the ratios of host to microbial DNA found in the milk samples. With respect to the mock community, it is possible that the discrepancy between observed and expected species ratios was due to a similar bias across methods, however this could not be determined in this study.

Overall, this evaluation has addressed two important problems in metagenomic sequencing of low diversity environments, specifically poor sequencing economics and poor microbial sequence depth. The results show that the host depletion approach of the ML kit performed better than the enrichment or direct sequencing alternatives by providing the potential for deeper strain level analysis without an observable bias. Our study additionally highlighted the importance of the correct downstream bioinformatic tool selection to accurately characterize the milk microbiome.

## Methods

### Collection, storage and initial treatment of milk samples

Five bovine raw milk samples and five human milk samples that were previously frozen at − 80 °C were thawed for use in this study. Bovine raw milk samples were obtained from farms across Ireland, taken from bulk milk tanks and transported to the laboratory on ice where a subsample was stored at − 80 °C. Human milk samples were collected as part of the Microbe Mom study, in accordance with the relevant guidelines and regulations, following informed approved consent of the mothers. These samples were collected within the first month after delivery. The maximum possible volume of available samples was used in the study, which was 30 ml of bovine milk and 4 ml of human milk. For one bovine and one human milk sample, an additional aliquot was taken for use as positive controls through spiking with a mock community. Milk samples were prepared as follows: bovine milk and human milk samples were centrifuged at 4500×*g* for 20 min at 4 °C. After centrifugation, the cream and supernatant were discarded and the remaining pellets were subjected to two washing steps, where the pellets were resuspended in sterile PBS and centrifuged at 13,000×*g* for 1 min, after which the supernatant was discarded. After the washing steps, the pellets were resuspended in 2 ml sterile PBS (Fig. 1). For the positive control milk samples, the pellets were resuspended in 1.8 ml of sterile PBS and then spiked with 200 µl of a mock community (see below for further details) and mixed thoroughly to ensure homogenization. All 12 samples were divided into two 1.5 ml tubes with 1 ml each for use in the subsequent DNA extractions (Fig. 1).

### Description of mock community (positive control) and negative controls utilised

The 10 Strain Even Mix Whole Cell Material (ATCC MSA-2003; ATCC-LGC Partnership, Middlesex, United Kingdom) was used in this study as a positive control. This mock community is composed of approximately 2 × 10^7^ ± 1 log cells of 10 strains, i.e., *Bacillus cereus, Bifidobacterium adolescentis, Clostridium beijerinckii, Deinococcus radiodurans, Enterococcus faecalis, Escherichia coli, Lactobacillus gasseri, Rhodobacter sphaeroides, Staphylococcus epidermidis* and *Streptococcus mutans*, at equal proportions). After reconstitution following the supplier’s instructions, the mock community was spiked into each of one human and one bovine milk sample as positive controls prior to DNA extraction (BMS12 and HMS12). Sterile PBS was used as a negative control for each of the methods.

### Host depletion, microbial enrichment, and DNA extraction

For use of the DNeasy PowerSoil Pro kit (PS), samples (250 µl) were processed according to the manufacturer’s instructions (Qiagen, West Sussex, United Kingdom) with the exception that the lysis step was performed using a TissueLyser II (Qiagen) for 10 min at 30 Hz and DNA was eluted at 50 µl and stored at − 20 °C. In the case of the MolYsis complete5 kit (ML), samples (450 µl) were processed according to manufacturer’s instructions (Molzym GmBH & Co. KG, Bremen, Germany). Briefly, host cells were lysed through the addition of a chaotropic buffer and the released nucleic acids were degraded by an enzyme, MolDNase. Microbial cells were then sedimented and lysed using reagents and proteinase K. Microbial DNA was then isolated and extracted using spin columns and 100 µl of DNA was eluted and stored at − 20 °C. For the NEBNext Microbiome DNA Enrichment kit (NEB), 25 µl of input material was obtained from DNA extracted and eluted using the DNeasy PowerSoil Pro kit. Based on the DNA concentrations (ng/µl), the corresponding amount of beads needed was calculated and used according to the manufacturer’s instructions (New England Biolabs, Massachusetts, United States). DNA was eventually eluted to 25 µl and stored at − 20 °C.

### DNA library preparation

DNA was quantified using the Qubit double-stranded DNA (dsDNA) high sensitivity assay kit (Invitrogen). In samples where no detectable DNA was observed from extraction, a full 5 µl of neat sample was used, otherwise 5 µl of 0.2 ng/µl DNA was used. All samples were prepared for shotgun metagenomic sequencing according to Illumina Nextera XT library preparation kit guidelines, with the use of unique dual indexes for multiplexing with the Nextera XT index kit (Illumina). Following indexing and clean up, libraries were pooled to an equimolar concentration of 1 nM and sequenced on an Illumina NextSeq 500 sequencing platform with V2 kit. DNA from samples extracted using PS underwent additional 16S rRNA amplicon sequencing according to the 16S metagenomic sequencing library preparation protocol (Illumina). DNA was amplified with primers specific to the V3–V4 variable region of the 16S rRNA gene and libraries were prepared from the extracted DNA. Samples were multiplexed with the Nextera XT index kit (Illumina), pooled to an equimolar concentration of 20 nM and sequenced on an Illumina MiSeq sequencing platform with a V2 kit. All sequencing was performed at the Teagasc Sequencing Facility, in accordance with standard Illumina protocols.

### Bioinformatic and statistical analysis of shotgun metagenomic data

Raw metagenomic shotgun reads were quality checked and trimmed with Cutadapt (v. 1.18) and FastQC (v. 0.11.8). Reads were then aligned to the bovine and human genome to determine the number of host reads using Bowtie2 (v. 2.3.4), all non-host reads were assumed to be microbial. For sub-sampling, paired non-host reads were rarefied to 50,000 reads five times, with seeds 3, 29, 50, 64, and 87, using seqtk (version 1.3). MetaPhlAn2, Kaiju, and Kraken2 were used for taxonomic classification and were compared against each other^37,39,42,43^. The NCBI nr/nt database was used for Kaiju and Kraken2*.* Bracken was used on the Kraken2 output to determine and compute taxonomic abundances^44^. Functional profiling was performed using HUMAnN2, with genes classified based on Gene Ontology domains^45^. Metagenome-assembled genomes (MAGs) were assembled using MEGAHIT, followed by binning using MetaBAT2 and quality-checking using checkM^46^. Kaiju was used in the taxonomic classification of MAGs. High-quality bins were classified as > 90% complete with < 5% contamination as suggested by Bowers et al.^27^. For 16S rRNA amplicon sequencing, data was processed as previously described^47^ and taxonomy was assigned using BLAST against the SILVA SSURef database release 132. All further bioinformatics analyses, data visualisation and statistics were done in R (version 3.6.3)^48^ using vegan, ggplot2 and pheatmap packages.

### Ethics approval

Ethics for collection of human breast milk samples was granted by the Research Ethics Committee of the National Maternity Hospital as part of the Microbe Mom clinical trial, registration number ISRCTN53023014. Samples were collected following informed written consent, according to relevant guidelines and regulations.

## Supplementary Information

Supplementary Information.

## Data Availability

The dataset generated and analysed during the current study are available in the ENA repository under accession number PRJEB38099.
