# Supplementary material for: Evaluation of methods for the reduction of contaminating host reads when performing shotgun metagenomic sequencing of the milk microbiome
Source: Sci Rep. 2020 Dec 10;10:21665. doi: 10.1038/s41598-020-78773-6 (PMC7728742; doi:10.1038/s41598-020-78773-6)
Supplement: Supplementary file 1 — Supplementary Information. [file 41598_2020_78773_MOESM1_ESM.pdf]

Evaluation of methods for the reduction of contaminating host reads when performing shotgun metagenomic sequencing of the milk microbiome

Min Yap[1,2] ^, Conor Feehily[1,3] ^, Calum J. Walsh[1,3], Mark Fenelon[1], Eileen F. Murphy[4], Fionnuala M. McAuliffe[3,5], Douwe van Sinderen[2,3], Paul W O'Toole[2,3], Orla O'Sullivan[1,3], Paul D. Cotter[1,3]*

[1]Teagasc Food Research Centre, Moorepark, Fermoy, Co. Cork, Ireland

[2]School of Microbiology, University College Cork, Ireland

[3]APC Microbiome Ireland, Cork, Ireland

[4]Precision Biotics, Cork, Ireland

[5]UCD Perinatal Research Centre, School of Medicine, University College Dublin, National Maternity Hospital, Dublin, Ireland.

^ Both authors contributed equally to this study

* Correspondence to Paul.Cotter@teagasc.ie

Supplementary Information

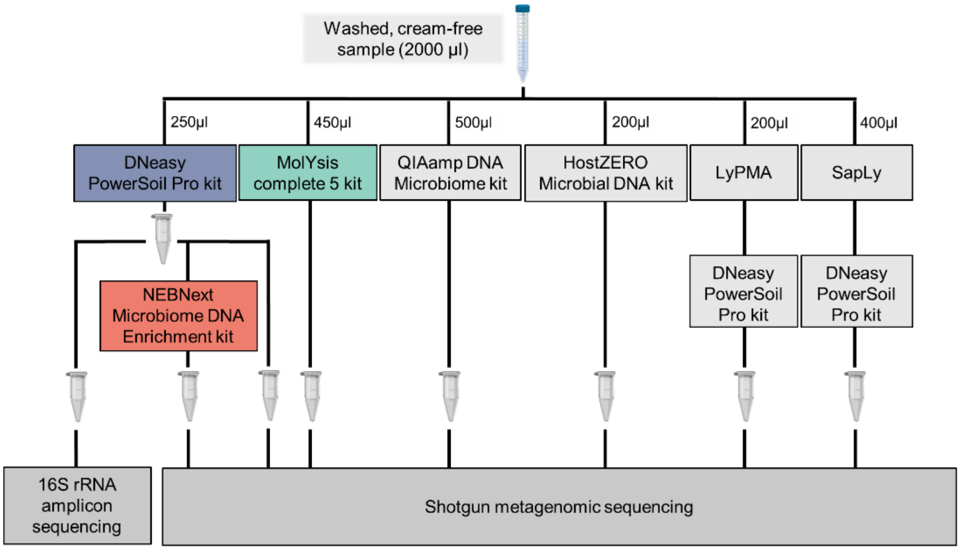

Supplementary Figure S1. Initial extraction plan. Two millilitres of the cream-free milk sample were to be divided into varying volumes depending on sample volume input recommended by the different kits and methods. The QIAamp DNA Microbiome kit and HostZERO Microbial DNA kit were both available commercially (Qiagen and Zymo Research). Both kits selectively lyse host cells prior to extraction of DNA from bacterial cells. Host depletion and subsequent DNA extractions were done according to the manufacturer's instructions for both kits. The LyPMA method, according to Marotz et al. (2018), involves osmotic lysis of host cells followed by PMA treatment to remove non-microbial cells in the sample. The SapLy method, according to Charalampous et al. (2019), uses a detergent to lyse host cells followed by enzymatic removal before further extraction of DNA from microbial cells. Further evaluation of the methods in grey were discontinued due to the limited starting sample volumes. This figure was created in part with BioRender.com.

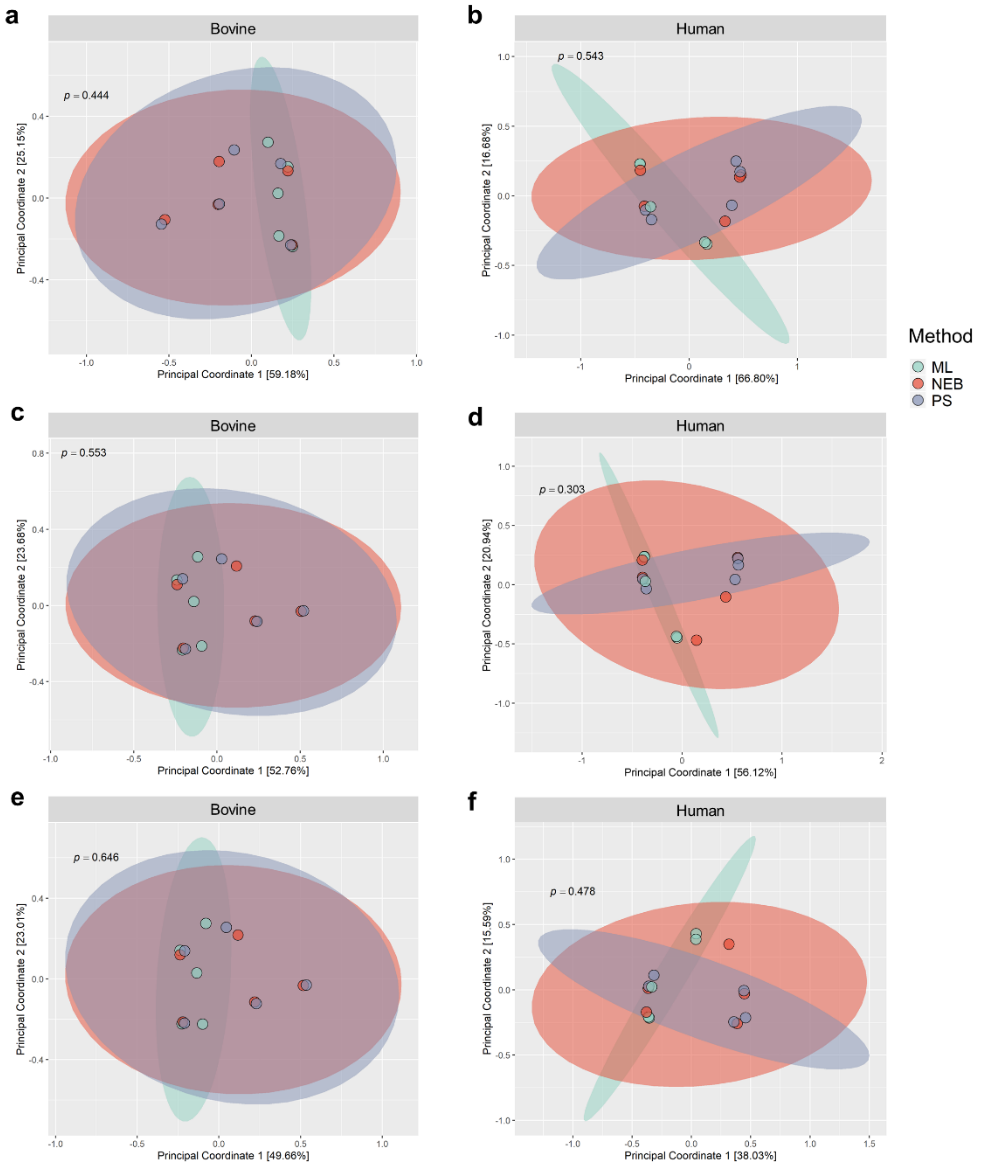

Supplementary Figure S2. Functional analysis of bovine and human milk samples as based on gene ontology domains. (**a**) Gene families related to cellular components for bovine and (**b**) human milk samples. (**c**) Gene families related to biological processes for bovine and (**d**) human milk samples. (**e**) Gene families related to molecular function for bovine and (**f**) human milk samples. Figures were produced using R[48].

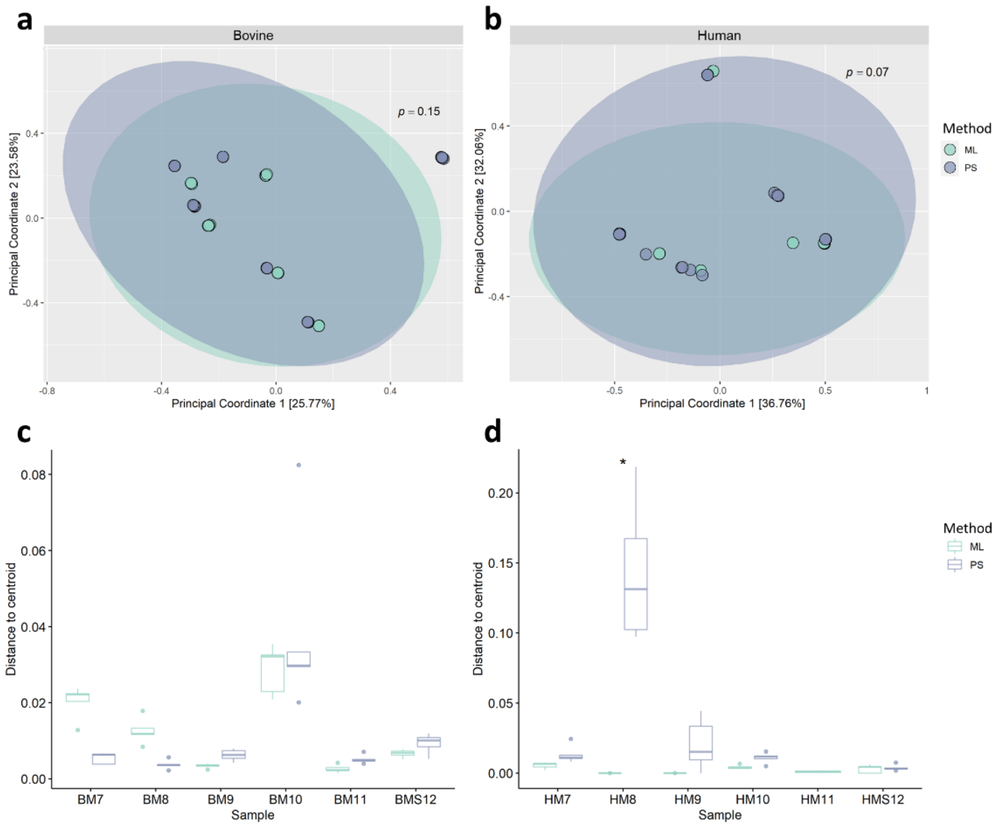

Supplementary Figure S3. Community composition and within-sample differences for samples subsampled to 50,000 non-host reads. Bray-Curtis dissimilarity plots for microbial communities from ML and PS kits were determined for (**a**) bovine and (**b**) human milk samples. Statistical community dissimilarities were calculated using ADONIS. The distances of samples to their respective centroids for each kit for (**c**) bovine and (**d**) human samples. Statistical differences were calculated using One-way ANOVA with post-hoc Tukey HSD test, with significant differences of a p value < 0.05 denoted with an asterisk. Figures were produced using R[48].

Supplementary Table S1. Summary of sequencing quality control data for each sample.

Sample	DNA yield (ng/μl)	Total number of raw reads	Total number of quality-filtered reads	Total number of non-host reads
BM7_ML	1.22	4,803,678	4,799,422	414,123
BM8_ML	2.02	4,218,166	4,213,627	521,433
BM9_ML	0.418	5,831,258	5,826,847	5,108,499
BM10_ML	0.764	5,503,695	5,489,219	1,559,305
BM11_ML	1.87	3,752,539	3,749,575	2,597,792
BMS12_ML	12	3,961,530	3,956,586	2,714,533
HM7_ML	0.134	4,577,558	4,572,083	801,292
HM8_ML	0.304	367,496	366,997	13,288
HM9_ML	< 0.001	899,823	898,330	22,347
HM10_ML	0.155	14,301,289	14,289,863	9,427,921
HM11_ML	< 0.001	2,558,808	2,555,194	51,285
HMS12_ML	4.01	3,135,085	3,128,637	2,913,481
PBS_ML	< 0.001	8,145	8,136	8,136
BM7_NEB	3.74	4,634,478	4,630,852	47,889
BM8_NEB	0.389	4,623,058	4,619,434	61,189
BM9_NEB	2.92	5,145,897	5,142,366	2,141,081
BM10_NEB	2.43	4,384,900	4,378,312	114,853
BM11_NEB	0.468	5,620,335	5,616,578	2,050,519
BMS12_NEB	2.4	2,990,318	2,987,713	588,814
HM7_NEB	2.17	3,714,043	3,711,382	118,695
HM8_NEB	2.86	3,505,245	3,502,584	76,561
HM9_NEB	< 0.001	7,449,515	7,444,889	137,544
HM10_NEB	1.54	4,046,734	4,043,758	166,690
HM11_NEB	< 0.001	4,773,599	4,770,318	124,328
HMS12_NEB	1.73	3,251,920	3,248,935	1,059,091
PBS_NEB	< 0.001	4,804	4,791	4,791
BM7_PS	16.6	4,306,883	4,298,577	53,485
BM8_PS	1.4	4,466,519	4,462,271	54,412
BM9_PS	12.2	949,504	948,705	174,822
BM10_PS	5.32	5,057,336	5,045,050	140,571
BM11_PS	1.93	4,039,654	4,036,626	511,785
BMS12_PS	5.83	3,734,020	3,730,408	712,548
HM7_PS	4.49	4,779,699	4,775,683	166,226
HM8_PS	5.45	3,434,585	3,431,627	97,069
HM9_PS	0.409	4,432,677	4,429,588	132,241

HM10_PS	4.08	4,606,083	4,598,639	201,712
HM11_PS	0.207	3,626,184	3,623,049	111,156
HMS12_PS	4.03	3,604,484	3,600,859	1,090,248
PBS_PS	< 0.001	659	654	654

Supplementary Table S2. List of species (> 1% relative abundance) assigned to negative control samples by each classifier

Classifier	PBS_ML	PBS_NEB	PBS_PS
MetaPhlAn2	*Lactobacillus crispatus*<br>*Aerococcus viridans*	No species assigned	No species assigned
Kraken2	*Aerococcus viridans*<br>*Cutibacterium acnes*<br>*Lactobacillus amylovorus*<br>*Actinoalloteichus* sp. AHMU CJ021<br>*Klebsiella pneumoniae*<br>*Lactobacillus johnsonii*<br>*Aerococcus urinaeequi*<br>*Negativicoccus massiliensis*<br>*Escherichia coli*<br>*Enterococcus faecalis*<br>*Rhodobacter sphaeroides*<br>*Acinetobacter johnsonii*<br>*Moraxella osloensis*	*Cutibacterium acnes*<br>*Propionibacterium* sp. oral taxon 193<br>*Pseudomonas pseudoalcaligenes*<br>*Micrococcus luteus*	*Pseudomonas alcaliphila*<br>*Negativicoccus massiliensis*
Kaiju	*Staphylococcus aureus*<br>*Aerococcus viridans*<br>*Lactobacillus crispatus*<br>*Escherichia coli*	*Staphylococcus aureus*	*Ralstonia solanacearum*<br>*Pseudomonas* sp. 286<br>*Acinetobacter nosocomialis*<br>*Enterobacter cloacae*<br>*Clostridioides difficile*<br>*Acinetobacter johnsonii*
